# Growth, Challenges, and Solutions over 25 Years of Mectizan and the Impact on Onchocerciasis Control

**DOI:** 10.1371/journal.pntd.0003507

**Published:** 2015-05-14

**Authors:** Joni Lawrence, Yao K. Sodahlon, Kisito T. Ogoussan, Adrian D. Hopkins

**Affiliations:** Mectizan Donation Program, Decatur, Georgia, United States of America; George Washington University, UNITED STATES

## History and Background

The Mectizan Donation Program (MDP) was established in 1987 to oversee Merck’s donation of Mectizan (ivermectin, MSD) for the control of onchocerciasis (river blindness) worldwide [1]. This was accelerated and expanded when Merck made a groundbreaking announcement in 1987: it would donate ivermectin, completely free of charge, as much as needed and for as long as needed, for the elimination of river blindness as a public health problem in all endemic countries.

P0rior to the donation of Mectizan, vector control was the only strategy used to control the disease. The World Health Organization’s Onchocerciasis Control Program (OCP) led the effort for 25 years, which resulted in very low endemicity in some West African countries. When Mectizan was donated, the implementation of mass drug administration (MDA) emerged as the primary strategy to control and eliminate onchocerciasis. The success of MDA led to the concept that drugs for other neglected tropical diseases (NTDS) could be distributed using the same strategy.

When MDP began, there was no distribution mechanism to reach the entire population at risk for onchocerciasis, particularly those living in remote, rural areas with poor health infrastructures. Merck approached a number of United Nations and international development agencies to request help distributing the drug, but because of lack of an established model, none came forward to facilitate distribution of the drug [2]. Determined to get the drug to the people who needed it, Merck established an independent program (MDP) and expert committee to develop a distribution mechanism to ensure the drug was distributed in a medically responsible manner with thorough supervision and monitoring [3]. The Mectizan Expert Committee (MEC) is made up of individuals with a broad range of expertise in global health, tropical diseases, entomology, parasitology, and disease control. The MEC meets twice-yearly and includes participation by WHO Headquarters, WHO Africa Regional Office (AFRO), the African Program for Onchocerciasis Control (APOC), the Onchocerciasis Elimination Program for the Americas (OEPA), the World Bank, and the Centers for Disease Control and Prevention (CDC).

The Onchocerciasis Control Programme (OCP) in West Africa began coordinating Mectizan distribution in some of the highly endemic areas for onchocercal blindness, while non-governmental development organizations (NGDOs) worked with Ministries of Health to cover the remaining areas of Africa. As mapping activities expanded, showing the real extent of the disease, it was clear that NGDOs and Ministries of Health did not have the resources to scale up as required. As a result, the Ministries and NGDOs, together with support from WHO and World Bank, created the African Program for Onchocerciasis Control (APOC) to coordinate distribution efforts throughout Africa.

**Fig 1:**
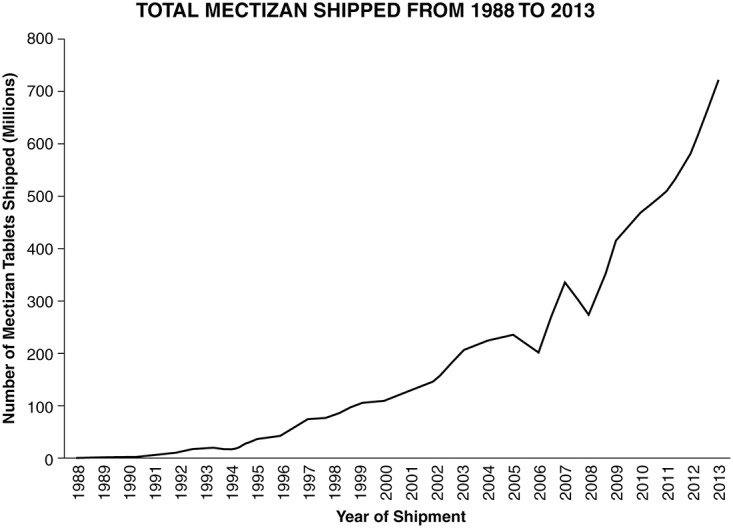
Number of Mectizan tablets (3 mg or equivalent) shipped from 1988 to 2013.

Mectizan for onchocerciasis is donated to Ministries of Health, mostly through NGDOs, and distributed by country programs and NGDO partners primarily through the Community Directed Treatment with ivermectin (CDTi) strategy, which was developed as a sustainable and cost-effective method to deliver Mectizan by the WHO Special Programme for Research and Training in Tropical Diseases (TDR) and APOC [4]. APOC adopted the CDTi strategy in 1997 and, by 2000, the Program and its partners reached more than 20 million people in 14 countries [5]. Today, APOC, its member countries, and NGDO partners reach up to 100 million people annually [6]. Between 1987 and 2013, enough Mectizan was shipped to implement more than 1.3 billion treatments for onchocerciasis in Africa, Latin America, and Yemen (see Fig 1). The term “Community Directed Interventions (CDI)” is now used to describe other interventions led by the communities built on the CDTi model. The success of partnerships created around the Mectizan donation led to other drug donations by other pharmaceutical companies, many of which are now donated through WHO.

## Operational Challenges

In addition to drug donations, MDP also provides technical and often additional financial resources to address specific problems encountered on the ground. The new CDTi strategy, distributing drugs at the community level, created some significant challenges (see Table 1). Mechanisms to determine where to treat evolved rapidly over a period of just a few years. Initially, cluster-based population studies were carried out using skin snips, an invasive technique not welcomed by communities. To address this issue, a non-invasive technique called “Rapid Epidemiologic Assessment” (REA) was developed. REA measured the disease burden by palpating onchocerca nodules in communities. The number of people in the community with nodules bore a fairly consistent relationship with skin snips to measure prevalence. TDR further developed the assessment tool and developed Rapid Epidemiological Mapping for Onchocerciasis (REMO), which was used in combination with REA studies in targeted communities based on the location of breeding sites of the black fly disease vector so that these two tools could be implemented to assess the disease burden based on ecology and geography [7]. Significant blindness was found only in hyper-endemic and meso-endemic areas. These areas were selected as priority areas for treatment to achieve the initial objective to control the disease.

**Table 1:** Overcoming challenges to Mectizan distribution.

Challenge	Solution
Lack of a distribution and oversight mechanism	1987: Merck establishes the Mectizan Donation Program and the Mectizan Expert Committee.
Insufficient NGDO support to scale up distribution across Africa	1995: WHO, the World Bank, Ministries of Health, and NGDO partners establish the African Program for Onchocerciasis Control (APOC).
Disease mapping (delineation of areas to be treated)	1992: Cluster-based skin snip surveys replaced by Rapid Epidemiological Assessments (REA), based on nodule surveys in each village, and then further adapted in 1995 to Rapid Epidemiological Mapping for Onchocerciasis (REMO), based on epidemiology and the ecology of the disease, which helped establish priority treatment areas.
Procuring and transporting scales for weight-based dosing	2003: Studies were conducted to develop height as a surrogate for weight and dose poles based on height were developed.
Dose and packaging was cumbersome for use in mass distribution	2003: Merck reformulated Mectizan from 6 mg to 3 mg tablets and replaced foil strips with bottles of 500 tablets to facilitate dosing and distribution in the field.
Short shelf life	2003: Merck re-evaluated stability, and shelf life was extended by one year.
Serious adverse events following Mectizan distribution in loiasis-endemic areas	2004: MEC/APOC Technical Consultative Committee (TCC) guidelines established. Research ongoing to develop strategies for onchocerciasis and lymphatic filariasis elimination in these areas.

There were challenges to implementing mass distribution of a drug with dosage based on weight. At the community level, weighing each patient to calculate the dosage was costly and cumbersome. Procuring and maintaining accurate scales was an issue, and they did not work well on uneven and dusty surfaces. Following a study in Nigeria, it was determined that height could be used as a surrogate for weight to determine the dosage [8]. Communities created a simple dose pole marked by height and the corresponding number of tablets, which solved the problem and is still used today for MDA for onchocerciasis and other NTDs.

The early packaging and tablet size of Mectizan (6 mg tablet) also created problems. First, the drug was packaged in foil strips of six tablets each that were cumbersome to open by community distributors in the field administering the drug to hundreds of people at a time. Second, the 6 mg tablet size presented a problem when it was necessary to split tablets to administer the correct dose. The NGDOs brought these problems to Merck’s attention and Merck reformulated the drug into a 3 mg tablet size packaged in bottles of 500 instead of the foil strips.

Merck further adapted to problems identified in the field by analyzing the drug’s stability, which resulted in extension of the shelf life from two to three years to prevent wastage and the need for disposal in areas where safe drug disposal was limited [9].

In 1998, Merck expanded the donation of Mectizan to include the elimination of lymphatic filariasis (LF) in countries co-endemic with onchocerciasis. The rapid scale up of treatments for LF required a significant increase in Mectizan production, which has been sustained since the expansion, although sufficient geographic coverage for LF elimination in Africa remains problematic.

In areas where loiasis is co-endemic with onchocerciasis and/or lymphatic filariasis, serious adverse events (SAEs) following treatment with Mectizan can occur in subjects with high *Loa loa* microfilarial loads. Loiasis is endemic in Central Africa and therefore presents a safety challenge because of the potential for neurologic SAEs, the cause for which remains to be fully elucidated [10]. MDP, MEC, APOC, Merck, the Bill & Melinda Gates Foundation, and other partners have worked diligently to find a solution to this issue. The MEC and APOC’s Technical Consultative Committee collaborated on a set of guidelines for program managers for safer implementation of CDTi in onchocerciasis and loiasis co-endemic areas, which have improved the outcomes of patients with SAEs [11]. Further scientific research is ongoing to determine the pathology of these SAEs, as well as the means to prevent them.

## The Future

By the end of 2012, the distribution of Mectizan had ceased in almost all endemic foci in the Americas. Colombia was the first country worldwide to receive verification of elimination of transmission by WHO [12]. The success in the Americas is due to the high geographic and therapeutic coverage achieved in limited and geographically isolated foci. This high coverage was achieved due to the political will in each country and the leadership demonstrated by OEPA.

There is increasing evidence that elimination can also be achieved in Africa with changes to the current control strategies in some areas, such as multiple yearly treatments and improved geographic and therapeutic coverage [13,14]. A number of factors will need to be considered, including improved integration of onchocerciasis and lymphatic filariasis efforts in co-endemic countries.

Drug forecasting needs to be much more accurately assessed to ensure that Merck’s manufacturing capacity is in sync with the demand. Now that Mectizan for onchocerciasis is available to almost all communities that need it, the average number of tablets needed each year is well known. However, some areas will increase the frequency of treatment from once to twice yearly as new strategies develop, and some hypoendemic communities will require treatment. Projections for scaling up Mectizan and albendazole distribution for LF elimination are also well estimated; however, implementation of the scale up in many countries has been slow to progress.

The success of Mectizan for onchocerciasis control and elimination can be attributed to the partnerships that have developed as a result of the donation. APOC has played a critical role in the distribution of Mectizan for onchocerciasis control and is now shifting its focus towards elimination. It will also focus on greater coordination with other health interventions for NTDs, beginning with improving the linkages between onchocerciasis and lymphatic filariasis and the necessary support to health services at the local and national levels. A number of African countries have worked with APOC and WHO AFRO to develop national master plans for NTD control. As APOC transforms into a new entity (provisionally named the Programme for Elimination of Neglected Diseases in Africa, or PENDA) at the beginning of 2016, new strategies will be developed for the elimination of these two diseases and for improved coordination of interventions for other NTDs. While this is a positive step forward, there will be new challenges, given competing obligations, priorities, and limited resources of health systems.

PENDA will urgently need to acquire the necessary financial and human resources to expand its role to further integrate interventions for multiple diseases that share similar strategies and target populations. Rapid scale up before 2016 is vital if elimination targets are to be met. The need for accurate forecasting and reporting will be essential during this time to avoid under- or, more likely, over-estimation of drug needs in each country. Over-estimation may result in excess inventory in storage-limited situations, causing a high risk of drug expiration, and in potential problems with manufacturing capacity.

Operational research needs are assessed by partners on an ongoing basis. Challenges remain to develop the most effective use of Mectizan to achieve elimination of both diseases in areas co-endemic for onchocerciasis, LF, and loiasis. Questions around the integration of interventions for NTD control need to be resolved. More research is needed to answer questions on the most efficient way to map diseases, identify priority treatment areas, coordinate MDA and other delivery systems, and monitor for safety [15,16].

## Conclusion

There have been many lessons learned during more than 25 years of Mectizan donation. MDP initiated the use of mass drug administration as a major strategy to control and eliminate river blindness. Donations from Merck have made the elimination of onchocerciasis a realistic goal for low-income endemic countries. Since the inception of the program, river blindness has been virtually eliminated in the Americas. The commitment of Merck remains firmly in place, and by 2025, there is hope that the donation will have achieved much more than was ever expected 25 years ago.
